# Real time PCR analyses of expression of E-cadherin, alpha-, beta- and gamma-catenin in human breast cancer for predicting clinical outcome

**DOI:** 10.1186/1477-7819-6-56

**Published:** 2008-06-11

**Authors:** Amit Goyal, Tracey A Martin, Robert E Mansel, Wen G Jiang

**Affiliations:** 1 Department of Surgery, School of Medicine, Cardiff University, Cardiff, UK

## Abstract

**Background:**

The E-cadherin catenin system acts as an invasion suppressor of epithelial malignancies. However, it is debatable whether expression of E-cadherin or catenins is a useful prognostic marker in invasive breast cancer.

**Methods:**

We measured the expression of E-cadherin and catenins (α-, β-, γ-catenin) in human breast carcinomas using real time quantitative polymerase chain reaction (Q-PCR) and investigated whether the expression levels were associated with known tumour variables or patient survival (median follow-up 72.2 months). RNA from frozen sections of breast tissue (tumour n = 124, background normal tissue n = 33) was reverse transcribed, quantified and analysed by Q-PCR with results expressed as number of copies of transcript/50 ng RNA.

**Results:**

There was no statistically significant difference in the expression of E-cadherin and catenins (α-, β-, γ-catenin)in the 33 paired normal background and tumour tissues. The expression of E-cadherin, α-, β-, and γ-catenin in node positive tumours was similar to node-negative tumours. E-cadherin, α-, β-, and γ-catenin expression in breast tumours was not related to Nottingham Prognostic Index (NPI). There was no significant difference in the expression of E-cadherin, α-, β-, γ-catenin between the various TNM stages. None of the molecular markers significantly influenced survival. Lymph node status was the only significant predictor of survival.

**Conclusion:**

Using real time quantitative PCR there was no difference in the expression of E-cadherin, α-, β-, γ-catenin between tumour and normal breast tissue. Furthermore, measurement of expression of these molecules was not of prognostic value in predicting long term outcome of women with breast cancer.

## Background

Development of malignant tumours is in part characterized by the ability of a tumour cell to overcome cell-cell adhesion and to invade surrounding tissue. The E-cadherin catenin complex localized to actin-based adherens junctions plays a crucial role in epithelial cell-cell adhesion and in the maintenance of tissue architecture. Perturbation in the expression or function of this complex results in loss of intercellular adhesion, with possible consequent cell transformation and tumour progression.

The main structure of E-cadherin catenin complex consists of transmembrane E-cadherin, cytoplasmic proteins, called catenins (α-, β- and γ-catenin), and actin cytoskeleton filament. β-catenin and γ-catenin share approximately 65% sequence homology and bind directly to the cytoplasmic tail of E-cadherin in a mutually exclusive manner. α-catenin then links the bound β- or γ-catenin to the actin microfilament network of the cytoskeleton[1,2].

In several carcinomas including gastric, head and neck, bladder, prostate, colon, and breast, a reduced or absent expression or abnormal location of E-cadherin has been observed [3-8]. Studies on both cell lines and clinical materials have provided evidence for the involvement of E-cadherin in suppressing cancer progression[5,9-13]. However, there are conflicting reports as to the usefulness of E-cadherin expression as an independent prognostic marker in invasive breast cancer [14-20]. In general, retention of E-cadherin expression correlates with well-differentiated, good prognosis and non-invasive properties[21]. Junctional catenin expression is often lost in cadherin-negative breast cancer and changes in catenin phosphorylation may compromise adhesion in cadherin-positive cancers[20,22-26]. However, few studies have investigated simultaneously the expression of E-cadherin, α-, β-, and γ-catenin. The expression of the various molecular markers in previous studies has been assessed by immunohistochemical staining in a small sample size. The use of immunohistochemistry does present some drawbacks in that staining interpretation may be somewhat subjective. It is also difficult to compare different series with different cut-offs for positivity and negativity.

We determined the expression of E-cadherin, α-, β- and γ-catenin in human breast carcinomas using real time quantitative polymerase chain reaction and investigated whether the expression levels are associated with known tumour variables or patient survival.

## Methods

### Patients and tumour specimens

Tumour tissue and normal background tissue (tissue away from the primary tumour site and histologically confirmed to be free from cancer cells, from the same patients) were collected with ethical approval and informed consent from patients with invasive breast cancer. The fresh tumour and normal background tissues were snap frozen in liquid nitrogen and stored at -70°C.

An overview of the clinical and pathological characteristics is summarised in Table 1. Follow-up information was obtained from the patient records at the hospital and the median follow-up for patients still alive was 72.2 months (at the time of these analyses). At the beginning of the project, all the samples were re-visited histologically by a consultant pathologist (ADJ, Department of Pathology, Cardiff University) to confirm the histology, nature and quality of tissues, as well as tumour/stroma ratio in each tissue. These samples were subsequently used for immunohistochemical staining and extraction of genetic material.

**Table 1:** Clinicopathological characteristics

**Variable**	**n**
Tissue type	
Normal background	33
Tumour	124
Tumour grade	
1	24
2	42
3	58
Histology	
Invasive ductal	94
Invasive lobular	14
Other	16
Stage (TNM)	
I	70
II	40
III	7
IV	4
Unknown	3
NPI^a^	
1 (good prognosis)	68
2 (moderate prognosis)	38
3 (poor prognosis)	16
Unknown	2
Outcome	
Disease free	85
Metastatic disease	7
Local recurrence	5
Death (breast cancer related)	15

^a^NPI, Nottingham Prognostic Index

### Real time-quantitative polymerase chain reaction

The levels of E-cadherin, α-, β- and γ-catenin transcripts from the prepared cDNA were determined by real time quantitative RT-PCR, based on the Amplifluor™ technology, using the method previously reported[27] Specific primer pairs for E-cadherin, α-, β- and γ-catenin (Table 2) were designed by the authors using a Beacon Designer software (version 2, CA, USA) and manufactured by Invitrogen (Invitrogen Life Technologies, Paisley, Scotland, UK). An additional sequence, known as the Z sequence (5'actgaacctgaccgtaca'3 as underlined in Table 2), which is complementary to the universal Z probe (Intergen Inc., England, UK) was added to one of the primer in the primer pair. Each reaction included Hot-start Q-master mix (Abgene), 10 pmol of specific forward primer, 1 pmol of reverse primer (with the Z sequence), 10 pmol of FAM-tagged probe (Intergen Inc.), and cDNA from approximate 50 ng RNA. An icyclerIQ™ (Bio-Rad) system, equipped with an optical unit that allows real-time detection of 96 reactions was used to amplify the plasmid standards and breast tissue samples under the following conditions: 94°C for 12 minutes; 50 cycles of 94°C for 15 s, 55°C for 40 seconds and 72°C for 20 seconds. The purified plasmids served as internal standards and helped in calculating the level of each tight junction molecule cDNA (copies per 50 ng RNA) in the tissue samples. The products of Q-PCR were verified on agarose gels.

**Table 2:** Primer pairs used for real time quantitative-PCR analyses.

***Primers for human α-catenin***	
ACATENINF1	caacccttgtaaacaccaat
ACATENINZR	actgaacctgaccgtacaccttctccaagaaattctca
	
***Primers for human β-catenin***	
BCATENINF8	agggattttctcagtccttc
BCATENINZF	actgaacctgaccgtacacatgccctcatctaatgtct
	
***Primers for human E-Cadherin***	
ECADF8	cagaaagttttccaccaaag
ECADZR	actgaacctgaccgtacaaaatgtgagcaattctgctt
	
***Primers for human γ-catenin***	
gCatF1	aacaagaacaaccccaagtt
gCatZr	actgaacctgaccgtacatagttacgcatgatctgcac

### Antibodies

The primary antibodies used were monoclonal anti-E-cadherin (HECD-1, mouse IgG1 1:50, R&D systems, Oxon, UK), anti-α-catenin (rabbit IgG1 1:50 dilution; Sigma), anti-β-catenin (mouse IgG1 1:100, R&D systems, Oxon, UK) and anti-γ-catenin (mouse IgG1 1:100, Sigma).

### Immunohistochemical staining

Immunohistochemical staining was performed on 25 matched tumour and normal background tissue pairs. Frozen sections of breast tumor and normal background tissue were cut at a thickness of 6 μm using a cryostat. The sections were mounted on Super Frost Plus microscope slides, air-dried and then fixed in a mixture of 50% acetone and 50% methanol. The sections were then placed in 'Optimax' (Vector Laboratories Ltd, Peterborough, UK) wash buffer for 5–10 min to rehydrate. Sections were incubated for 20 min in a 0.6% BSA blocking solution and probed with the primary antibody. Following extensive washings, sections were incubated for 30 min in the secondary biotinylated antibody (Multilink Swine anti-goat/mouse/rabbit immunoglobulin; Dako Inc.). Following washings, Avidin Biotin Complex (Vector Laboratories Ltd) was then applied to the sections, followed by extensive washings. Diaminobenzidine chromogen (Vector Laboratories Ltd) was then added to the sections, which were incubated in the dark for 5 minutes. Sections were then counterstained in Mayer's haematoxylin and dehydrated in ascending grades of methanol before clearing in xylene and mounting under a coverslip.

### Quantitative image analysis of immunohistochemical stains

#### Image acquisition and processing

Slides were viewed using a 20 × 20 objective lens (Leitz, DM IRB) and images were subsequently saved as a 24 bit colour JPEG file image via a digital camera (Panasonic, digital), and computer (Pentium III, RM machines, Milton Keynes UK) equipped with a frame grabber (Win TV, Celebrity Edition from Hauppauge). The captured images of tumour and normal background tissues were amalgamated using the PHOTOMERGE option in Adobe Photoshop 6. The images were then converted into gray scale images and inverted using Adobe Photoshop 6 before analysing using Optimas image analysis software (Version 6, Optimas, UK).

The intensity was analysed using point morphometry. 10 representative points were marked on each image captured. Overall, optical intensity data (mean and SD) was calculated by summing up the data from all images in the two groups and subtracting the mean background reading.

### Statistical analysis

The data obtained was analysed using the MINITAB 13.32 (Minitab Inc. State College, PA, USA) programme. Statistical significance was calculated using the two-sample student t-test, non-parametric Mann-Whitney test and ANOVA where appropriate. Multivariate analysis was done for survival.

## Results

The intensity of membrane staining for E-cadherin and all catenin molecules was significantly more in normal background tissues compared with tumour tissues (mean ± SD; E-cadherin normal background 169.6 ± 5.83, tumour 82.7 ± 12.78 p < 0.001; α-catenin normal background 163.22 ± 4.27, tumour 92.22 ± 21.02 p < 0.001, β-catenin normal background 216.1 ± 15.94, tumour 99 ± 32.93 p < 0.001, γ-catenin normal background 131.9 ± 24.99, tumour 85.5 ± 29.93 p = 0.008).

In contrast, no statistically significant difference was seen in the expression of E-cadherin, α-catenin, β-catenin and γ-catenin in the 33 paired normal background and tumour tissues (copies/50 ng RNA, mean ± SD: E-cadherin normal background 17.4 ± 3.8, tumour 16.5 ± 6.7 p = 0.51; α-catenin normal background 13.5 ± 4.5, tumour 38.3 ± 30.3 p = 0.48, β-catenin normal background 0.048 ± 0.029, tumour 0.057 ± 0.019 p = 0.68, γ-catenin normal background 1.255 ± 0.927, tumour 0.219 ± 0.157 p = 0.28).

The expression of E-cadherin, α-, β- and γ-catenin in node positive tumours was similar to node-negative tumours (copies/50 ng RNA, mean ± SD: E-cadherin node positive 35.5 ± 104.2, node negative 25.70 ± 35.13 p = 0.51; α-catenin node positive 26.60 ± 61.79, node negative 17.25 ± 23.08 p = 0.84, β-catenin node positive 0.0973 ± 0.2003, node negative 0.0895 ± 0.1686 p = 0.69, γ-catenin node positive 0.635 ± 4.004 node negative 0.622 ± 1.948 p = 0.55).

There was no significant relationship of E-cadherin, α-catenin, β-catenin and γ-catenin in breast tumours to Nottingham Prognostic Index (NPI) (E-cadherin p = 0.094, α-catenin p = 0.144, β-catenin p = 0.378, γ-catenin p = 0.131).

There was a trend towards decreased E-cadherin expression in Grade 2 and 3 tumours compared to Grade 1 tumours but the differences were not statistically significant. α-catenin expression was significantly increased in Grade 2 tumours compared with Grade 1 tumours (p = 0.03). However, α-catenin expression in Grade 3 tumours was similar to Grade 1 tumours. β-catenin expression was similar in Grade 1 and 2 tumours. However, its expression was significantly increased in Grade 3 tumours compared to Grade 2 tumours (p = 0.054). γ-catenin expression was similar in the 3 groups.

Surprisingly, there was no difference in the expression of E-cadherin catenin complex between ductal and lobular tumours.

The TNM Stages 3 and 4 were combined into a single group for analyses as they were very small. There was no significant difference in the expression of E-cadherin, α-, β-, and γ-catenin between the various TNM stages (p = 0.282, p = 0.806, p = 0.838, p = 0.337 respectively).

E-cadherin expression in tumours of patients who were disease free was significantly more compared to those with metastatic disease, local recurrence or dying from breast cancer (Disease free vs. poor outcome (metastatic disease and/or local recurrence and/or death from breast cancer) p = 0.012). There was no difference in α-catenin and γ-catenin expression between the groups. β-catenin expression was increased in patients dying from breast cancer compared to disease free patients and the difference approached statistical significance (p = 0.052). Multivariate analysis was done using SPSS for mortality. None of the molecular markers significantly influenced survival. Lymph node status was the only significant predictor of survival.

## Discussion

In this study, we examined the expression of cell-cell adhesion molecules E-cadherin, α-, β- and γ-catenin in human breast cancer by quantitative real time polymerase chain reaction. The mRNA levels of these markers were related to clinicopathological variables and survival data.

The data from this study did not show any difference in the expression of E-cahderin, α-, β- and γ-catenin between tumour and related normal tissue. This contrasts with the results of immunohistochemical staining in the present study and most previously reported studies of E-cadherin catenin complex in breast cancer, which have described down-regulation of some of these molecules in tumourigenesis[20,23-26,28].

The disparity can be easily explained as this is the first study to measure expression of the E-cadherin catenin complex molecules by quantitative real time polymerase chain reaction. Direct comparisons are not possible as previous studies have varied widely in patient samples and immunohistochemical scoring methods, which make comparisons difficult.

It is possible that a defect in the E-cadherin catenin complex without a change in its expression may be responsible for the malignant progression. Immunohistochemical staining and Q-PCR reveal different information and each has its advantages and disadvantages. Immunohistochemical analysis provides vital information on the protein location in the cells, which is important when studying cell adhesion molecules. However, the method has obvious limitations in quantifying the true amount of the protein in cells or tissues. Quantitative analysis of mRNA as presented here has the unique advantage in providing quantitative information of the gene expression concerned. However, this method does not provide information about the location of the molecule within a cell and may be considered 'over-sensitive'.

The expression of E-cadherin, α-, β- and γ-catenin was similar in node positive and node negative tumours. Our results suggest that expression of E-cadherin, α-, β- and γ-catenin may persist into the later stages of breast carcinoma. Siitonen et al. and Oka et al. found a correlation between loss of E-cadherin and the presence of nodal metastases[29,30], but this has not been widely reported. Howard et al. recently reported increased E-cadherin expression in tumour tissue with nodal metastases[14]. E-cadherin expression is retained in inflammatory breast cancer[31]. Furthermore, derivative metastases frequently show strong E-cadherin expression[32]. One emerging opinion is that dynamic, reversible modulation of E-cadherin catenin complex occurs during breast carcinoma progression. E-cadherin catenin complex expression or function is transiently reduced at the development stage of primary tumours. This loss of adhesiveness at primary site of tumours allows cancer cells to 'dissociate' from each other. However, following invasion and degradation of surrounding matrix, and migration into the vasculature and surrounding tissue, E-cadherin catenin complex is re-introduced and cells adhere to the vasculature and form tumour emboli[14].

In contrast to most previous IHC studies [33-35], mRNA expression of E-cadherin, α-, β- and γ-catenin was similar in ductal and lobular tumours. This may be due to the fact that quantitative analysis as given here reflects the total amount of the molecule rather than in an individual cell and that mRNA expression does not always correlate with cellular protein expression. Moreover, most series reported on membrane staining and did not include cytoplasmic staining as a separate category. Thus, it is possible that lobular carcinoma cases with cytoplasmic staining were included in the general category of reduced expression of E-cadherin staining.

E-cadherin expression was not associated with tumour grade. Reduced E-cadherin expression has been associated in the past with high histological grade[19,36]. Our results suggest that this is not necessarily the case. Preserved or increased E-cadherin catenin expression supports the notion that it assists aggressive tumour growth by providing a support structure for cells to adhere and accelerates invasion and metastasis.

There are conflicting reports in the literature regarding the relationship between E-cadherin catenin complex and prognosis/survival. In this study, E-cadherin catenin complex expression was not prognostic in breast cancer patients or related to survival. Asgeirsson *et al*., and Heimann *et al*., reported reduced expression of E-cadherin to be associated with tumour recurrence, metastases, and poor prognosis in breast cancer[15,16]. Not all studies confirm these findings[14,19,37]. Other investigators have shown that the abnormal expression of catenins is related to poor prognosis or decreased survival[20,23,26,38].

Despite the apparent advantages of quantitation and sensitivity, the use of quantitative real time polymerase chain reaction does present some drawbacks. The technique is highly sensitive and any contaminating cells (lymphocytes, stromal tissue) will lead to readings that belong to non cancer cells. To overcome this problem, it is essential to isolate pure populations of tumour cells by micro dissection.

## Conclusion

In conclusion, using real time quantitative PCR we have shown that there is no difference in the expression of E-cahderin, α-, β- and γ-catenin between tumour and normal breast tissue. Furthermore, measurement of expression of these molecules is not of prognostic value in predicting long term outcome of women with breast cancer.

## Competing interests

The authors declare that they have no competing interests.

## Authors' contributions

AG conducted the study, analyzed the data and prepared the manuscript, TAM contributed to the conduct of the study, REM contributed to clinical follow ups and helped in editing the manuscript, WGJ contributed to the conduct of the study, design of primers and statistical analysis.
